# *“I didn’t take it too seriously because I’d just never heard of it”:* Experiential knowledge and genetic screening for thalassaemia in the UK

**DOI:** 10.1002/jgc4.1042

**Published:** 2018-12-24

**Authors:** Felicity K. Boardman, Rachel Hale

**Affiliations:** ^1^ Warwick Medical School Coventry UK

**Keywords:** experiential knowledge, genetic screening, reproductive decision‐making, social and ethical implications, thalassaemia, UK

## Abstract

Members of the public face particular challenges when undergoing reproductive genetic screening. Lack of family history with genetic disease has been identified as a key barrier affecting screening uptake and responses to genetic risk. This study explores this obstacle using beta thalassaemia as a case study. Fifteen in‐depth qualitative interviews were conducted exploring the reproductive views and decisions of people at risk of transmitting thalassaemia. Eleven participants had thalassaemia themselves and/or were members of an affected family. Four participants were identified as thalassaemia carriers through genetic screening programmes with no family history. Notable differences were observed between these two groups. For thalassaemic individuals and families, past experience clarified and facilitated their sense of reproductive responsibility, however carriers struggled to relate to, and incorporate the information into their lives. It was witnessing their child becoming symptomatic—rather than receiving a diagnosis or genetic risk information per se that had the most substantial influence on carriers’ subsequent views and decisions. Educational resources used to support genetic screening programmes would benefit from an engagement with the experiential accounts of life with genetic disease in order to more effectively bridge the chasm in knowledge and understanding between affected families and the general public, towards whom expansive genetic screening is aimed.

## 1 INTRODUCTION

As new reprogenetic technologies—such as those used to undertake whole genome sequencing—are becoming increasingly subsumed within standard National Health Service care, the possibility of their use as a population‐level genetic screening tool is now being considered in earnest, both within and beyond the UK (Hasegawa, Fergus, Ojeda, & Au, 2011; Tarini & Goldenberg, 2012). Indeed, the perceived centrality of genomics to the future of medicine is reflected by its high position on the UK public agenda (Davies, 2016) and the documented widespread interest, and investment, in genomic medicine by the UK general public (Middleton et al., 2016).

When considering the management of relatively common genetic diseases, many of which continue to lack effective treatments, this shift towards population‐level genomics is significant. While individually rare, when viewed together, the risk to the health of the public posed by such genetic disorders becomes more substantial (Archibald et al., 2018). Indeed, recent research suggests that when combined, the prevalence of the three most common genetic disorders (Cystic Fibrosis, Fragile X syndrome and Spinal Muscular Atrophy [SMA]) is comparable to that of Down's Syndrome (Archibald et al., 2018)—a condition routinely screened for within standard NHS antenatal care. This prevalence, when considered alongside the unpredictable and variable prognoses associated with many of these genetic disorders and their relatively 20 limited treatment options all contribute to the mounting case in favour of offering carrier screening programmes to the general public (Gregg et al., 2014; Nazareth, Lazarin, & Goldberg, 2015; Plantinga et al., 2016).

Although identifying carriers of genetic conditions in the general population in either the pre‐conceptual or prenatal period confers particular opportunities for the carrier couples it identifies—primarily through the extension of their (currently limited) reproductive options (HGC, 2011)—this approach also brings with it practical, ethical and social dilemmas that may surface in genetic counselling contexts.

One persistent challenge identified in the literature relates to the receptivity of the general population to the notion of their potential carrier status (Archibald et al., 2009; Beard, Amor, Pietro, & Archibald, 2016; Ioannou, Delatycki, Massie, Hodgson, & Lewis, 2015; McClaren, Delatycki, Collins, Metcalfe, & Aitken, 2008). As NHS carrier testing has typically only been reserved for families already known to be affected by genetic disease, those undergoing carrier testing have usually approached the decision in the context of their prior knowledge of the condition—a factor known to have a significant influence on reproductive decisions and attitudes (Baillergeau & Duyvendak, 2016; Etcehgary et al., 2008; Kay & Kingston, 2002; Raspberry & Skinner, 2010; Ziebland & Herxheimer, 2008). Indeed, research data have revealed key differences in the reproductive attitudes and decisions between those who have (and those who lack) prior experience of the condition being tested for (Boardman, Young, Warren, & Griffiths, 2017; Etchegary et al., 2008). Those with direct experience have been found to typically view the condition in a more favourable light than those who lack this form of insight (Boardman, Young, Warren, et al., 2017; Watson, Williamson, & Chapple, 1991), although this has been shown to vary according to the nature and impact of the condition (Kay & Kingston, 2002). Through familiarity with the challenges and opportunities associated with a particular condition, experiential knowledge may instil confidence in a person's perceived ability to cope with a child with that same condition (Boardman, 2014). By acting as a “window” into potential futures, this intimate way of knowing and experiencing genetic disease allows for the realistic envisioning, and appraisal, of different reproductive outcomes (Boardman, 2014; Etchegary et al., 2008; Markens, Browner, & Preloran, 2010) in a way that may be more challenging to those for whom genetic disease is a more removed and abstract concept (Archibald et al., 2009).

When experiential knowledge of genetic disease becomes dislocated from the reproductive decisions that concern it, however, through the practice of genetic screening, there are significant implications not only for reproductive attitudes, but also uptake of carrier screening in various contexts (Archibald et al., 2009, 2018; Ioannou et al., 2014; McClaren et al., 2008). Plantinga et al.’s (2016) study of public attitudes towards the offer of a carrier screen for 50 genetic disorders, for example, demonstrated that the most common reason for lack of interest in undergoing the screen was “not wanting to be bothered” by the knowledge that one could be a carrier for genetic disease (Plantinga et al., 2016p. 5). Similarly, Prior et al.’s (2010) study of carrier screening for SMA highlighted the most common reason for screening refusal among the 500 pregnant or pre‐conceptual women offered an SMA screen was reported as a lack of concern about SMA. Lack of family history, already being a parent to healthy children, not being of advanced maternal age and viewing oneself as fit and healthy have all been identified as informing perceptions of the chances of being a genetic carrier (and consequently screening uptake) in spite of the irrelevance of these factors to actual genetic risk status (Beard et al., 2016; McClaren et al., 2008). Even for the most prevalent genetic diseases, public awareness and understanding of the nature of genetic inheritance remain demonstratively poor (Anido, Carlson, Taft, & Sherman, 2005; Braido et al., 2015; Fanos, Spangner, & Musci, 2006; Moultrie, Kish‐Doto, Peay, & Lewis, 2016; Watson et al., 1991) with interest in carrier screening correspondingly low. The only exception to this trend is instances where the test is offered or recommended directly by a health care professional (Rothwell, Anderson, Swoboda, Stark, & Botkin, 2013).

The perception that genetic disease is only relevant to the sub‐set of the population already living with them is further reflected in the reactions of shock and unpreparedness experienced by carrier couples in the wake of positive screening test results (Anido et al., 2005; Beard et al., 2016; Ioannou et al., 2015). Unlike families already living with genetic disease, screening‐identified carrier couples need to quickly absorb large quantities of complex information about the condition they carry and/or make difficult decisions about pregnancy (dis)continuation, often within a compressed timeframe, presenting significant challenges to genetic counsellors (Ioannou et al., 2015).

Within the UK, the carrier screening programme for thalassaemia is currently the only prenatal genetic screening programme offered within standard NHS care. All pregnant women are screened for thalassaemia carrier status, usually before they reach 10 weeks gestation, and if found to be a carrier, are invited to have the father of the foetus tested and/or diagnostic testing of foetus, depending on the results.

Thalassaemia refers to a group of inherited recessive blood disorders that affect the production of haemoglobin within the body, affecting approximately 70,000 newborns annually and making them among the most common single trait recessive disorders worldwide (Cousens, Gaff, Metcalfe, & Delatycki, 2010). Approaches to thalassaemia screening vary drastically in the international arena, with mandatory pre‐marital screening programmes underway in countries with particularly high prevalence (e.g., Iran, Saudi Arabia, Palestinian Territories and Cyprus) (Cousens et al., 2010). Thalassaemia carriers (also known as people with thalassaemia “trait” or thalassaemia “minor”) typically do not have any serious associated health problems (although may be mildly anaemic), but have the potential to pass thalassaemia on to any future children, if conceived with another thalassaemia carrier. When two thalassaemia carriers reproduce, each child born to them has a one in four chance of having thalassaemia.

The primary treatment for beta thalassaemia is regular (every 4–6 weeks) blood transfusions to treat and prevent anaemia, usually in conjunction with chelation therapy to treat any associated iron build up. The current average life expectancy (with treatment) of a person diagnosed with thalassaemia today is between 40 and 50 years old, but this is expected to increase significantly overtime as treatments improve (Telfer, 2009).

Whilst only one carrier screening programme is currently implemented at population level in the UK, shifts in the capacities of genomic technologies suggest that this model of screening provision is likely to expand in the future. Increasing numbers of would‐be parents will be called upon to make decisions about the conditions they would want to know their carrier status for, and which they would not, often without any experience or knowledge of the conditions around which they are making such decisions (Plantinga et al., 2016). It is on account of this unfamiliarity with the world of genetics that proponents of pre‐conception genetic screening have highlighted the need for high quality information and support to provide the infrastructure for any screening programmes implemented (Prior et al., 2010), as well as a need to better understand the role and value of prior experience with genetic disease within reproductive decisions (Boardman, Young, Warren, et al., 2017).

In order to address this under‐explored topic, this paper offers an analytic comparison of the views, experiences and reproductive decisions of 15 people at risk of transmitting thalassaemia with contrasting levels of prior experience and knowledge of the condition. Eleven participants approached their decision in the context of a family history with thalassaemia (i.e., either they had thalassaemia themselves or a member of their family did), and four participants made their reproductive decisions having been identified as a thalassaemia carrier through antenatal or newborn genetic screening, but without any prior history of, or experience with, the disease. The contrast between these two positions is illuminated in order to offer critical insight into the role that these disparities of knowledge and experience have on reproductive views and decisions, before considering their policy and practice implications.

## 2 METHODS

This study involved in‐depth qualitative interviews with 15 participants who either had prior experience of thalassaemia, or encountered it through antenatal genetic screening.

## 3 RECRUITMENT

Participants were recruited into the study through the largest support group for thalassaemia in the UK, the UK Thalassaemia Society (UKTS). The UKTS offers support to people across the UK who are either carriers of thalassaemia, have thalassaemia themselves or have a diagnosis of thalassaemia in the family. Three separate calls were placed in the society's e‐mail delivered publication between June and December 2017, requesting that potential participants get in touch with the project researcher.

## 4 INTERVIEWS

Upon initial contact with the study team, participants were provided with an information leaflet outlining the aims of the research and what their participation might involve. They were then asked to contact the researchers to arrange an interview if they remained interested in being involved. After this point, participants were asked to sign a consent form, and interviews were carried out either face‐to‐face or over the telephone, depending on participant availability, health needs and preference. The majority of face‐to‐face interviews were carried out in participants’ homes although one was conducted in the participant's workplace and another in a public space within a hotel (Table 1).

**Table 1:** Participant characteristics

Participants	Numbers	Gender		Ethnicity		Interview type		Thalassaemia identification route	
		Female	Male	Asian	European	Face‐to‐face	Telephone	Screening	Family history
Diagnosed with thalassemia	8	5	3	5	3	3	5	0	10
Parent of person diagnosed with thalassemia	7	5	2	7	0	2	5	4	1
Totals	15	10	5	12	3	5	10	4	11

The interview schedule was developed by reference to the relevant literature, as well as the lead researcher's previous work in this area (Boardman, 2014; Boardman, Young, & Griffiths, 2017; Boardman, Young, Warren, et al., 2017). It covered participants’ prior experiences with thalassaemia, their perceptions of quality of life, any reproductive decisions made or anticipated, views on selective reproduction and views and attitudes towards population genetic screening specifically for thalassaemia, as well as for other conditions. The interviews lasted on average for 43 min, with telephone interviews averaging 39 min duration and face‐to‐face interviews 49 min. The range of the interviews was between 21 and 74 min. All interviews were audio‐recorded and transcribed verbatim by a professional transcription service (with names and identifiers removed).

## 5 DATA ANALYSIS

A modified grounded theory approach was undertaken to analyse the resulting data. Initially, one researcher conducted “open coding” (Gibbs, 2007) which was largely descriptive, before more discriminatory hierarchical coding was undertaken using qualitative data analysis software, Nvivo 11. Both researchers were involved in hierarchical coding through a process of coding and refinement of concepts (through data interpretation, reference to the literature and regular analysis meetings between the researchers). Discrepancies in coding were discussed between the researchers and following agreement, re‐coding was carried out until “theoretical saturation” (i.e., no new concepts were emerging) had occurred.

## 6 PRESENTATION OF RESULTS

Although 15 participants were interviewed and an analysis of all of their accounts contributed to the thematic framework, four participants have been selected for detailed presentation within this paper. These four accounts were selected on the basis of their particularly eloquent representation of the views and experiences of other participants within the two groups (those who had prior experience with thalassaemia or those who encountered it though antenatal/newborn genetic screening) Moreover, as the concept of experiential knowledge is so critical to this analysis, a focus on the narratives of four participants allows for a more detailed and nuanced exploration of participants’ stories and their relationship to their reproductive decisions and attitudes, which would not have been possible in detail for all 15 participants. To protect the identities of the four participants, all names appearing within this paper are pseudonyms.

## 7 ETHICAL APPROVAL

Ethical approval for the research was granted by The University of Warwick's Biomedical and Scientific Research Ethics Committee on 4th April 2017 (REGO‐2017)

## 8 RESULTS

The calls for participants resulted in contact from 21 people which in turn led to the successful completion of 15 interviews. Six people who initially made contact with the research team were not interviewed as attempts to arrange an interview were unsuccessful. After two attempts to arrange an interview with each person, further contact was abandoned. One participant contacted the team for interview after the completion of the analysis and was accepted for interview. Their transcript was analysed using the thematic framework developed from the previous interviews.

The 15 participants who went through for interview either had thalassaemia themselves, or had at least one person diagnosed with thalassaemia in their family (see Table 2). All of the four participants who were identified as thalassaemia carriers through genetic screening went on to have at least one child with thalassaemia.

**Table 2:** Participant experience with thalassaemia and screening

Participant number	Pseudonym	Age	Sex	Ethnicity	Religion/religious community	Parental status	Number of children with thalassemia	Nature of experience with thalassaemia	How thalassaemia was discovered
1	Bilal	46	M	Asian (Pakistan)	Muslim	✔	0/5	Has thalassaemia	Symptoms
2	Mahila	36	F	Asian (Lebanese)	Catholic		0	Has thalassaemia	Symptoms
3	Ameena	38	F	Asian (British Indian)	Muslim	✔	1/4	Carrier	Antenatal carrier screening
4	Giovanni	36	M	European (Italian)	Catholic		0	Has thalassaemia	Symptoms
5	Jahida	33	F	Asian (Pakistan)	Muslim	✔	3/3	Carrier	Antenatal carrier screening
6	Imran	45	M	Asian	Muslim	✔	0/5	Has thalassaemia	Symptoms
7	Hamid	25	F	Asian (Iranian)	Muslim		0	Has thalassaemia	Symptoms
8	Inas	36	F	Asian (Bangladesh)	Muslim		0	Has thalassaemia	Symptoms
9	Deeba	49	F	Asian (Pakistan)	Muslim	✔	1/2 (adopted)	Parent of adopted child	Symptoms
10	Chaaya	68	F	Asian (British Indian)	Hindu	✔	1/1	Carrier	Child's Symptoms
11	Jamini	52	M	Asian (British Indian)	Hindu	✔	1/2	Carrier	Child's Symptoms
12	Luna	44	F	European (Sardinian)	Catholic/Agnostic		0	Has thalassaemia	Symptoms
13	Marina	35	F	European (Greek Cypriot)	Greek Orthodox		0	Has thalassaemia	Symptoms
14	Fadwa	40	F	Asian (Pakistani)	Muslim	✔	1/2	Carrier	Newborn screening
15	Arjun	41	M	Asian (British Pakistani)	Muslim	✔	1/4	Carrier	Antenatal carrier screening

Participants ranged in age from 25 to 68, nine were parents and seven of these were parents to a child/children with thalassaemia, with one participant having multiple affected children (Jahida) (see Table 2). Only two of the nine parents had thalassaemia themselves (Bilal and Imran), both were male, and neither had gone on to have affected children themselves (Table 2). The vast majority of participants were female (10), and all were of South‐Asian, South‐East Asian or Mediterranean heritage, reflecting the typical ethnic prevalence of thalassaemia (Hickman et al., 1999). All participants described themselves as belonging to a religious community (see Table 2), however, four participants described this as reflecting their social identity rather than their personal beliefs. The participants were geographically dispersed throughout England.

The results have been divided into two sections depending on participants’ prior experiences with thalassaemia. First, the accounts of participants who considered their reproductive views through the lens of an established family or personal history with thalassaemia will be presented (*n* = 11), before contrasting these with the accounts of those who discovered the condition in their family through genetic screening (*n* = 4).

### 8.1 *‘I know exactly how hard it can become…’*: Experiential knowledge and reproduction in thalassaemic families

For the majority of participants within the sample (11), thalassaemia was a condition for which they had considerable experience to draw on as they considered their own reproductive views and decisions. Eight of these participants had thalassaemia themselves (with two, Giovanni and Imran, also having an affected sibling), and two were parents of an affected child who had not been identified prenatally (Jamini and Chaaya). For these participants, considering their own reproductive attitudes and decisions (both past and anticipated) was inextricably linked to the everyday reality of life with thalassaemia.

Imran was a 45 year old man at the time of his interview, and had been diagnosed with thalassaemia shortly after birth. He was living and working part‐time as a taxi driver in an English city, and had five children (all carriers of thalassaemia, but without the condition themselves) born through an arranged marriage that Imran described as having since broken down. Imran described thalassaemia as a sceptre that had overshadowed much of his life, contributing to him being “overlooked” and “written off” within his family. He described there being accepted belief within his family that he would not live past the age of 25, a view that when combined with his family's Muslim faith contributed to the pressure Imran felt to accept an arranged marriage:

I was pushed into an arranged marriage. And the excuses they were giving me at the time was I going to be dead by the age of 25, so you need to have… “get married and have children so that God is happy with you”. And it wasn't just my parents saying this, it was my brothers, and I have a big family. And then at the time I was working in my uncle's restaurant, so I was getting it from my uncle as well, so there was huge pressure, peer pressure, family pressure to just go along with it.

For Imran, becoming educated about his thalassaemia in his early 30 s (by joining support groups and meeting other people with the same diagnosis) and becoming more proactive in its management, as well as “coming out” as a gay man were critical turning points in his life. Up until that point, his life had very much been under the control of a heavily stigmatised and disabling condition that he did not fully understand and an influential family with very particular ideas about how best to offset the disadvantages his condition was seen to impose.

Indeed, it was Imran's overwhelmingly negative experiences with thalassaemia and the reactions to it of those around him that invariably influenced his views of it as a condition as well as his perceptions of reproductive morality:

I resent that I was born with thalassaemia actually…[…]… I've seen my fair share of suffering and I don't think parents should even be given the choice….[…]… you know…I think thalassaemia should be eradicated and the government should be forcing Asian parents to stop their children marrying within the family just for the property, and also to have screening before marriage, like they did in Greece, you know? If someone had… if two carriers, if two people are carriers they shouldn't be allowed to have children, simple as that. But if they have the pregnancy and there are stem cells or that kind of thing that can be done in the pregnancy then, fine, the baby can live, but if there's no cure while the baby's in pregnancy then I think it should be terminated, yeah. I think that's kinder….But I'm quite a bit radical, people probably would object to me being radical, but I'm sorry you don't know my life and as far as I'm concerned I don't wish this on anyone.

The attitudes of people with genetic disorders towards carrier or prenatal screening for the same disorder they have has been relatively under‐explored in the literature (Boardman, 2014), however, recent studies produce a somewhat contradictory picture, with some showing support, and others more ambivalent or negative attitudes towards reproductive genetic medicine (Barter, Hastings, Williams, & Huws, 2016; Janssens et al., 2016; Roadhouse et al., 2018). For Imran, thalassaemia, which he referred to as “the giant’ in his life, was at the root of much of the unhappiness, social exclusion and physical suffering that he described as marring his formative years. It was this intimate, first‐hand experience of the condition which he felt gave him both the authority—but also the authenticity (‘*I'm sorry but you don't know my life*’)—with which to promote state‐controlled approaches to genetic screening. However, not all participants” views were as clear‐cut as Imran's, with some viewing their detailed knowledge and experience with thalassaemia as more of a double‐edged sword in the context of reproductive genetics.

Mahila was 36 years old at the time of interview and living in an inner‐city area in England with her husband, Haneef. She was working full time for a charity and was, undergoing fertility treatment to have her first child. Mahila was diagnosed with thalassaemia at the age of 6 months in Lebanon, her country of birth and where she spent her first 20 years. She described her experience of growing up in Lebanon as “difficult” and “isolating” given the limited understanding around thalassaemia, the absence of contact with other children with the same diagnosis and the prevalent social stigma of genetic disease, which became more pronounced overtime. For Mahila, relationships were a key area where the stigma of her genetic disease was most keenly felt:

Relationship wise, in Lebanon with the Lebanese mentality, and I guess…mum and dad were not as aware of the taboo and the stigma, when I was very little….But…[…]…. at some point mum started getting a hint of, with the Lebanese mentality is she going to find… if she wants to get married is she going to find a man who is strong enough to say to his family, “this is what I want”? And I think she started thinking‐ probably not. And I didn't know at that point why she was telling me these things because I was quite young, but I remember being a teenager…and my mum telling me… “you don't need a man in your life, and if you want to have children there are ways to have children without a man.” Now I understand.

While not specifically asked about, the stigma of living with a genetic haemoglobinopathy was spontaneously mentioned by five of the 11 participants in this group, highlighting its significance in shaping experiences of the condition, particularly among particular ethnic groups where arranged marriages and family prestige are a central part of religious, cultural and social life (Roy & Chatterjee, 2007; Shaw & Hurst, 2006; van der Wal et al., 2015). Indeed, Mahila described her first boyfriend's family rejecting her as the potential long‐term partner for their son on account of her “defective gene” as an “awful” experience that informed her later attitudes towards relationships. Like Imran, Mahila described the people around her as having low expectations of her life, reinforced by her periods of frequent illness (which she described as increasing in frequency as she has gotten older) and her secondary infertility. Despite these experiences, however, and unlike Imran, Mahila still described thalassaemia as having a positive (and continuing) role in shaping her identity and personality:

….in some ways it has really helped me shape my personality. Because I was outside the general pack of teenagers I… yes, it was difficult, but at the same time I think it gave me the impetus to be me, and very early on I decided I think, I'm going to be very much an individual who makes her own decision, who doesn't follow societal norms, who… it gave me a lot of individualism. And I think you can see it in my life choices a little bit, you know, in Lebanon you… I was doing very well academically and when we do very well academically in Lebanon you try to go for medicine and try and go for legal…there's a certain expectation…[…]…And I went, “yeah I want to do charity work”, which is considered a weird choice for someone who was performing well academically. So I think that coming to terms with my difference early on in those teenage years kind of shaped me.

Shakespeare (2006), among others (Boardman, Young, Warren, et al., 2017; De Wolfe, 2002), have highlighted the contrast in outlook and attitude between disabled people who have fixed, static impairments (often present from birth) and those with acquired, fluctuating or degenerative impairments which typically involve periods of pain, suffering and illness (p. 106). He has argued that people who fall within the latter group are far more likely to experience their impairment as intrusive, burdensome and entirely separate from their sense of self and identity than those whose impairments are fixed and stable. Although thalassaemia might be considered a fluctuating impairment according to Shakespeare's categorisations, involving frequent periods of illness and medical treatment, Mahila's account demonstrates the way in which, though her condition varied, her experience of stigmatisation, of being a “tainted” person (Goffman, 1963) remained constant and impacted the contribution thalassaemia made to the constitution of her identity in numerous ways. By setting her apart from the norms and expectations of a typical Lebanese woman, Mahila felt empowered to develop her own sense of identity and self that she might not otherwise had the autonomy to do.

Viewing thalassaemia in this way (as simultaneously providing opportunities for individualism as well as the challenges of ostracisation and exclusion) and knowing the condition intimately, however, left Mahila with difficult decisions when approaching her own reproductive decisions. Her (now) husband, Haneef, underwent pre‐marital genetic screening for thalassaemia prior to their wedding and was found not to be a carrier, although Mahila described their lack of control over this screen, with it being conducted in Lebanon where such screening is a marital prerequisite. Mahila described her current attitude to thalassaemia screening in the following way:

….I kind of asked Haneef this two days ago I said, suppose we had a child and we knew, we tested it and we knew that the child had thalassaemia he said, “so you manage it, I would have probably gone for it.” So it's ambivalent right?...[…]….Thalassaemia is a difficult one I think because, because I have it and I've managed it and you go, ‘it's not that bad, it's bad…. it's not as bad as that’, so then you think, why go through an abortion really?…You know, it's not the end of the world and it's a manageable condition, and science is improving day after day. And so for us it's not maybe a good enough reason to let a pregnancy go. However, and having said all that, my first instinct would still be to have an abortion… because I know exactly how hard it can become.

Even as Mahlia's experiential knowledge served to reassure her that having a child with thalassaemia would not be the “end of the world” because she had “managed”, she simultaneously acknowledged the more difficult parts of life with thalassaemia “how hard it can become”, resulting in ambivalent and contradictory feelings regarding the potential transmission of thalassaemia to future generations. This finding is mirrored by the literature on attitudes towards screening among people with genetic/chromosomal disorders (Boardman, 2011; Boardman, Young, Warren, et al., 2017; Chen & Schiffman, 2000; Middleton, Hewison, & Mueller, 1998). Although Mahila's experiential knowledge gave her a unique standpoint from which to appraise the life of any future child with thalassaemia, she also acknowledged the limits on how far this knowledge could be transposed onto the lives of future generations in a meaningful way:

In my younger years, into my like twenties I was fine. I think it's now, in my thirties, that I've started realising that‐ and I hadn't realised this before‐ that as you grow older it actually becomes a little bit harder because your body is growing older…[…]…The most concerning aspect of thalassaemia for me – and I'm talking personally, not in general – personally it's just literally that I am making it up as I go along. I've not been through being 40 with thalassaemia, 50 with thalassaemia, 60 with thalassaemia‐ I don't know what's going to come, all I can do is try and take as good care of myself as I can, but that is definitely the most concerning aspect for me.

Although Mahila had previously described her experiential knowledge as being instrumental to the way she and Haneef approached reprogenetic decision‐making, allowing them to feel more comfortable with the idea of having a thalassaemic child than they might otherwise have been, her experiences of growing older with thalassaemia also highlighted to her the parts of the thalassaemia trajectory that Mahila's experiential expertise could not yet reach. The shifting nature of her experiences brought into critical relief the intrinsic limitations of this type of knowledge when used as a measure of disease severity and projected quality of life for future generations (Boardman, 2017). While on the one hand giving them privileged insight into the disorder, experiential knowledge also emerged from Mahila's account as a bounded form of insight, both limited, and contoured, by the unique set of circumstances through which it was generated.

Despite its inherent limitations, there was nevertheless evidence from all of the 11 participants who had direct experience of thalassaemia in their family that their experiences served as the yardstick by which future lives could be anticipated and appraised. For some of these participants, this insight both bolstered and authenticated their views and decisions (e.g., Imran), whether this be to prevent the recurrence of thalassaemia, or a belief that thalassaemia was an insufficient justification for the use of genetic technologies and pregnancy termination. However, for the remaining participants, experience of thalassaemia introduced new layers of ambiguity and uncertainty into their reproductive views, with some unclear how they would respond if faced with selective termination decisions. In these instances, being a member of an already affected family could serve to heighten existing tensions that surround prenatal screening and testing practices (Kelly, 2009). More specifically, the negotiation of the fine balance between managing a sense of genetic responsibility for future family members’ health and a desire not to express disvalue towards affected family members, a concern that has been identified in relation to various different genetic conditions within the literature (Kay & Kingston, 2002; Kelly, 2009; Raspberry & Skinner, 2010; Shakespeare, 2006).

Although participants’ experiential knowledge emerged as an influence capable of both clarifying and muddying the waters of selective reproduction, the remaining four participants in the sample however, approached their reproductive decisions from an entirely different vantage point. For these participants, thalassaemia was first encountered not through the tangible and visible processes of symptoms and diagnosis, but rather through the far more abstract pathway of genetic screening.

### 8.2 “With carriers, it's not a major thing is it? You just carry on normal…”: Prenatal screening, experience and reproductive decision‐making

Of the 15 participants in this study, four discovered the thalassaemia trait in their family through a genetic screening programme rather than through the illness and subsequent diagnosis of a family member. Three of these participants were informed of their carrier status following antenatal screening during their first pregnancy (Jahida, Arjun and Ameena), and one described discovering their child's thalassaemia following a heel prick test at birth (Fadwa). Of the three who discovered their carrier status through antenatal screening, two (Jahida and Arjun) had a first born child with thalassaemia.

One participant, Ameena, however, while discovering her carrier status (and that of her husband Sadeed) through antenatal genetic screening during her first pregnancy, declined all diagnostic testing and went on to have two unaffected girls before her third child, a son named Taysir (aged five at the time of interview), was diagnosed with thalassaemia at birth. Aged 38 at time of interview, Ameena was working full time and living with her husband, extended family and four children in the North‐West of England. Due to Ameena's work schedule and that of her husband, their extended family—especially Ameena's two younger sisters—were heavily involved in the care of the children.

In comparison to his three sisters (all of whom carry the thalassaemia trait but do not have the condition), Taysir's life was described by Ameena as “hard”, marked by high medical and support needs and regular (every 3–4 weeks) transfusions in hospital together with daily chelation therapy. Ameena described the monthly transfusions as particularly traumatic before Taysir had a permanent port implanted to facilitate access to his veins. In spite of this, however, thalassaemia treatment remained a difficult and taxing process for Taysir and the wider family as Ameena described:

…[…]…and then every night, because his iron is high, because he has regular blood transfusions, his iron builds up so we've got to do iron chelation every night. He's on Desferal, it's like injecting him every night in the legs. Now his legs are sort of bruised; we could do it on his stomach but he's got no fat there…So it's always on the legs, poor lad, he knows it's cream time when I have to put cream on and then after that poor kid, inject him. He's just so used to it, poor lad, and sometimes it does hurt him because sometimes I have to take the needle out because it's like swollen up for some odd reason, we don't know why. and then I put it in a different place. But… it's horrible.

Ameena stressed throughout her interview that thalassaemia is a “very serious” condition that negatively impacted all spheres of family life. She described not only the administration of his treatment regime, but also the side effects of it (osteoporosis and weakened tooth enamel) and the constant anxiety surrounding the possibility of him receiving contaminated blood via transfusion as all contributing to Taysir's ongoing poor health, the strains placed on family life and consequently her negative perceptions of the condition overall.

Despite articulating a clear view throughout her account that thalassaemia is a serious condition that involves implicit suffering, Ameena's intimate, in‐depth and ongoing experiences of having a child with thalassaemia were in stark contrast to her earlier understanding of the condition at the point of her first encounter with it, during her first pregnancy. Discovering both she and Sadeed were carriers through antenatal screening, Ameena acknowledged the lack of resonance with her life and family experiences that thalassaemia had at that time:

I'll be honest, you know when I got pregnant and they were saying, “you're a carrier, and he's [Sadeed, husband] a carrier and you might have a chance of having a major” [affected child], at that point in time I just didn't realise how severe it was or how it would affect me or my child, you know. I just fobbed it off like, ‘oh you know, they say a lot of things…’ and I didn't think much of it..[…]…And then my first two [children] were carriers, so I was like, ‘oh it's okay, you know, so like…’ and then he [Taysir, third child] turned out to be the major. And they told us when he was little….[…]…And even then it didn't sort of click to me and I thought, you know, okay, because I'd never read about it, I didn't know exactly what it was, I'd never seen anybody with thalassaemia, I didn't even know it ran in the families at all. Because some of them were abroad and I wasn't too much in contact with my dad, so I didn't know much about it. And then they did a blood test about two months old and they said he's really low in iron, we need to do his first blood transfusion, and I cried my eyes out. And that's when it hit me, yeah …that there's something severely wrong here.

Although the identification of carriers in the antenatal period has been heralded as expanding reproductive autonomy and choice for would‐be parents (Locock & Kai, 2008; Tsianakas, Atkin, Calnan, Dormandy, & Marteau, 2011), it can nevertheless be a frightening and bewildering experience for pregnant women and their partners (Beard et al., 2016; Ioannou et al., 2015; Locock & Kai, 2008). As many women are not aware that carrier screening for thalassaemia is taking place, or regard the screen as a compulsory component of high quality prenatal care (Cousens et al., 2010), the discovery of carrier couple status for a genetic condition completely unknown to them has been described as akin to entering a “new world” (McClaren et al., 2008) of genetic disease.

It was in the context of these liminal spaces that emerged through the discovery of carrier status that participants’ experiences and knowledge of health and illness gained significance in the formulation of their responses to their genetic risk (Archibald et al., 2009; McClaren et al., 2008). As an autosomal recessive disorder, each “at risk” pregnancy conceived by a carrier couple has a one in four (25%) chance of being affected by thalassaemia. In the face of this uncertainty, participants looked to their family histories and those of their friends and wider communities *(“I'd never seen anyone with thalassaemia before”*), together with own encounters with health and disease *(“I just always felt I was healthy and so would have healthy children”) *as well as their perceptions of their child's health to estimate the significance of that genetic risk. The fact that Ameena had never heard of thalassaemia before was pivotal to her estimation of the scale of the threat (“*If it was that bad, you'd know about it, wouldn't you?”*). Together with the fact that her first two daughters were born healthy carriers of the condition, Ameena's experiences bolstered her in her conviction that thalassaemia was not something to be overly concerned about—this was something she could “*safely ignore”*. It was upon witnessing Taysir's deterioration and his subsequent reliance on blood transfusions that Ameena felt the reality of the condition really “*hit home”*. It was in the context of this acquired experiential knowledge that Ameena approached her fourth pregnancy with revised understandings of her genetic responsibility, undergoing diagnostic testing and going on to have an unaffected (carrier) daughter.

Like Ameena, Arjun and Fadwa also opted to undergo prenatal diagnosis in subsequent pregnancies after experiencing thalassaemia directly through their first born child, both receiving clear results on those tests and going on to have three unaffected daughters and one unaffected son respectively. However, direct experience of thalassaemia did not change the reproductive decisions of all participants in this group in such a dramatic way as was observed with Ameena, Fadwa and Arjun.

Jahida was 33 years old at the time of her interview, a working mother of three children aged between 14 and nine, all of whom were diagnosed with thalassaemia at birth. Although Jahida was informed of her carrier status (and that of her husband, Parvez) during her first pregnancy, Jahida nevertheless felt the genetic risk to her children was minimal:

To be honest I never ever heard of it [thalassaemia] before. I only found out when I was first pregnant with my eldest one, I had a blood test and they told me at that point. That was the first time I actually heard of it, but to be honest I didn't really take much notice of it because I didn't think it was going to happen to me, I kind of thought, “oh, okay”. And they said it's one out of four chances. And I was quite young myself and because I'd never heard of it, I didn't research into it and it turned out to be, you know, that actually she was born with it…[…]…They did call us in to do a bit of counselling and went through this condition, but to be honest I still didn't take it seriously at all because I'd just never heard of it and I thought, one in four chance, I thought, well three chances it won't happen, so I was quite positive, but you never know.

Although Jahida's daughter, Aaleyah, was diagnosed with thalassaemia at birth, her symptoms remained relatively mild and she did not require blood transfusions until she was 4 years old. It was during this time of symptom stability and good health that Jahida and Parvez had their second child (Miras), opting not to use prenatal diagnosis:

I thought, “no, well Aaleyah was fine” and we were giving her a healthy diet and she was still managing, so we kind of went into that belief that she would be okay and it won't happen to us twice and she will probably just be severely anaemic and we just need to give her a little extra support but she won't need transfusions. So then obviously I had my son [Miras], but eventually after that Aaleyah went onto it [blood transfusions] at four years old, then a year later he went onto it.

Due to Aaleyah's relatively good health, Jahida's perception of thalassaemia was initially largely benign, a viewpoint she reported as being upheld by her husband and wider family. Indeed, despite a confirmed diagnosis of thalassaemia, the continuation of Jahida's conviction that *“it won't happen to us” *is striking. It was only after the initiation of blood transfusions for both of her children, Aaleyah and Miras, that Jahida's view of thalassaemia, and consequently her perception of the genetic risk to future family members, began to change. Jahida approached her third pregnancy with a profound sense of “genetic responsibility” (Kenen, 1994; Raspberry & Skinner, 2010) to prevent transmission of the condition, although ultimately her decision contrasted sharply with that of Ameena, Arjun and Fadwa:

…..By now[third pregnancy] I was worried yeah and I was offered a test [prenatal diagnosis, CVS test]…[…]…they said they had to like put a needle in and check it from the stomach and then they will tell me there and then. But they said “there's a risk of miscarriage”. So I prepared myself everything for it, went for the counselling, actually went on the day to the hospital. And I got there and I just changed my mind at that point because I think I was three months along and I just thought, “I can't do it, because what if it is healthy and I end up losing it?” So I was like in so many different minds that I couldn't carry on. And then when she was born and they told me [that she has thalassaemia] I was really upset again, but either way I wouldn't want to lose any of my children anyway, so I just carried on.

Authors such as Lippman (1999) and Katz Rothman (1986) have highlighted the burden of responsibility that the emergence of genetic technologies confers on pregnant women. The availability of such technologies, delivered under the rubric of standard antenatal care, expands ideals of responsible motherhood by subsuming within them responsibility for the (genomic) health of future generations. As Reed has noted, the boundaries of this “genetic responsibility” are contoured through the lens of racial and gendered politics, reflecting a congruence of inequitable relations beyond the domain of reproduction (Reed, 2011). For mothers of children with genetic disorders, such as Jahida, however, this sense of genetic responsibility took on very particular meaning and significance. As Kelly (2009) discovered, mothers of children with genetic disorders often consciously side‐step subsequent reprogenetic decision‐making so as not to be put in the fraught position of needing to choose between continuing with an affected pregnancy or aborting a foetus with the same condition as their existing child. For Jahida, declining the CVS test was ultimately not a rejection of her sense of reproductive genetic responsibility, but rather an alternative expression of it, whereby providing her child with the opportunity to live was prioritised over her felt responsibility to prevent thalassaemia.

For both Jahida and Ameena, therefore, as well as the other two participants in the sample who discovered their thalassaemia status through a screening result, experiential knowledge of thalassaemia was pivotal to the interpretation and processing of their genetic risk information. Without grounding in their everyday realities, their carrier screening results lacked meaning and context and were consequently disregarded, a situation which Ameena regarded as common:

….you know I think there could be a lot of women out there like me who don't pay much attention. I remember a few years back it was on TV and it used to be on so much, you know, adverts about it. But it never pointed, clicked to me or it didn't stand out to me until I had experience of it. So maybe if there was something out there that really sort of clicked with everyone and sort of made them aware of it, then I've just… Because if I'd have known before… oh I don't know, this is it, I'm still a bit confused about that…[…]….I just don't believe in abortion. But maybe if I'd have done it a different way, like IVF or something.

Although Ameena did, in fact, know her carrier status before having her four children, her assertion that she would have acted differently had she fully “known” and understood the experiential reality of life with genetic disease starkly highlights the dearth of accessible information on genetic disorders available to would‐be parents facing carrier screening results. Moreover, her account also draws attention to the intrinsic limitations of genetic risk information in the absence of an experiential “anchor” with which to ground the information in a person's everyday life.

## 9 DISCUSSION

As genetic technologies continue to advance in their sophistication and capabilities, there are calls to expand genetic carrier screening panels for increasing numbers of genetic conditions. It has been argued that such a move will accord prospective parents—especially carrier parents—more autonomy and control over their reproductive outcomes than has previously ever been possible (Henneman et al. (ESHG)., 2016). Such shifts, however, also entail the reconfiguration of ideals of responsible parenthood and justice as reproductive outcomes which previously fell under the auspices of chance or luck come to be considered controllable and amenable to human manipulation (Denier, 2014).

Acknowledgement of the high (combined) prevalence of genetic disorders, the decreasing cost and increasing ease of genetic screening, together with the dearth of effective treatments for even the most common genetic disorders have all served to heighten these calls for their prevention through screening programmes. However, within the UK, thalassaemia remains the only genetic condition for which a prenatal carrier screening programme exists. This study, to the best of our knowledge, presents the first analytic comparison between the perspectives of those who discovered their family's thalassaemia trait through a screening programme, and those whose reproductive views emerged from their everyday stocks of knowledge acquired through being part of an affected family. A consideration of the differences, but also the similarities between the perspectives of these two groups is critical as it highlights some of the implications when reproductive decision‐making is transferred from already affected families to members of the public through the use of genetic screening programmes.

For those participants whose reproductive views and decisions emerged from a rich familial or personal history with thalassaemia, experiential knowledge was critical to the way in which they formulated their perceptions of their genetic risk, envisaged future generations affected by thalassaemia and ascertained where their reproductive responsibilities lay. While for some participants (e.g., Imran) this sense of responsibility was first and foremost to prevent the transmission of thalassaemia to the next generation others presented more ambivalent views, conflicted by the inherent contradictions between their over‐arching sense of responsibility to future generations (to prevent genetic disease) and their daily experience of thalassaemia, which for many was described as *“not so bad”* (Mahila). First‐hand experience was constructed by all of these participants, therefore, as a privileged form of insight held by thalassaemic families (Mahila) that both improved the quality and authenticity of reproductive decision‐making, even as it could complicate the decisions made. Indeed, this was the case even in instances whereby its fallibility as a knowledge resource was acknowledged, or its usage led to ambiguity or conflicted understanding of parental responsibility.

For participants who discovered they were carriers of thalassaemia through prenatal or newborn genetic screening, however, perceptions of genetic risk and responsibility were constructed in entirely different ways. All four participants who discovered their carrier status in this way reported that their antenatal (or newborn) screening result was their first encounter with thalassaemia, having never heard of it before, nor met anyone with it. In the absence of this direct experience, this sub‐set of participants turned to a range of different resources to make sense of, and estimate the seriousness of, their genetic risk. Experiences with personal and familial health, of community and faith practices, the stories and views of family and friends together with internet resources and medical information were all drawn upon by this group to aid the interpretation and appraisal of their genetic risk. Ultimately, three out of four of these participants decided to proceed with their first pregnancies without further testing, a process that resulted in the births of two children with thalassaemia and one child with thalassaemia trait. As Etchegary et al. (2008) have observed, in the absence of “vivid” forms of knowledge about a condition (i.e., direct experience of the condition), people facing antenatal screening typically turn to more “vague” forms of experiential knowledge (such as the stories of unknown others) to make sense of their genetic risk. However, as demonstrated by the participants in this study, vague and vivid forms of knowledge were not equally valued, nor comparable in terms of their impact on reproductive views and decisions. Rather, these forms of knowledge were hierarchically ordered, with lived experience “trumping” all other forms of knowing. Indeed, for all four participants in this group, it was the onset of serious thalassaemia symptoms in their child (rather than the thalassaemia diagnosis itself, or the concomitant provision of medical information) that had the most profound effect in the re‐ordering of previously held beliefs and expectations of the condition. Experiential knowledge emerged as having the most significant, and enduring, impact on genetic risk perceptions, re‐calibrating participants’ sense of reproductive accountability, even as this accountability could lead to entirely polarised reproductive decisions and outcomes in practice (e.g., Jahida vs. Arjun).

## 10 CONCLUSIONS

Although uptake of thalassaemia carrier screening within the UK population is high, indeed it has been suggested that many pregnant women are not even aware it is even being carried out or consider it a mandatory part of prenatal care (Cousens et al., 2010), the reactions of shock and disbelief to positive screen results both within this study and the wider literature highlight the difficulties pregnant women and their partners have in assimilating into the world of genetic disease in the absence of prior experience of the condition. For Jahida and Ameena, for example, lack of family history with thalassaemia, ambivalence about pregnancy termination, positive perceptions of their own health and having had previously healthy children all contributed to the diminished sense of importance they assigned to their screening results and their perceived irrelevance to their lives. These reasons mirror those documented in the literature on screening refusal more broadly (Ioannou et al., 2014). It is noteworthy that for both women, the lack of resonance that their screening results had even persisted following the diagnosis of thalassaemia in their child. Indeed, it was only following the onset of symptoms (that were serious enough to require treatment) that significant shifts occurred in the perceptions of thalassaemia, and correspondingly, in participants’ estimations of their genetic risk and responsibility. This finding underscores the centrality of direct experience to the interpretation of, and reaction to, abstract ideas such as genetic risk. Experiential knowledge brought thalassaemia out of the realm of the hypothetical and abstract and into the everyday lives of these participants in a way that genetic risk statistics and diagnoses could not. Research into the reactions of members of the general population to carrier screening have highlighted the difficulties in absorbing and relating to medical information about a condition that one has not directly encountered (Archibald et al., 2009; Beard et al., 2016). As such, bringing genetic disease “into the worlds” of the general public who largely view it as the domain of small groups of affected families has been identified as a key challenge in the successful implementation of carrier screening programmes (McClaren et al., 2008).

## 11 PRACTICE IMPLICATIONS

This study highlights the particular difficulties facing people who discover their carrier status through genetic screening as opposed to through the symptoms and diagnosis of a family member. As has been described elsewhere in the literature, the participants who fell into this group faced unique challenges when assessing the relevance and significance of their results in the context of the rest of their lives (Archibald et al., 2009; Beard et al., 2016; Ioannou et al., 2012; Wright et al., 2015). This was particularly the case for participants who already had healthy children and who had never before heard of thalassaemia, but also those whose experience of the condition was limited to a very mildly affected or pre‐symptomatic child (e.g., Jahida).

The experiential knowledge of affected individuals and families is a significant, yet under‐utilised, resource that may be harnessed in the context of genetic counselling in order to address some of these difficulties. It has been suggested that insights from affected families could be imparted to genetic counselling patients through a variety of means; for example, through personal stories, photographs, vignettes, videos and interviews, all of which may assist in humanising and “bringing to life” a genetic condition in a way that the impartation of purely clinical information often fails to do (Ahmed, Bryant, & Hewison, 2007). Support and advocacy groups for already affected families may be a particularly important resource in developing and evaluating these resources. There is limited published data exploring the usage of these groups by individuals and couples identified as carriers, particularly those facing complex decisions about invasive diagnostic testing and/or selective pregnancy termination. For well‐established screening programmes, like that for Down's Syndrome for example, however, such avenues of support and information are well‐developed and used (Down's Syndrome Association, 2017). The lack of correspondingly well‐developed mechanisms of information and support within the groups for genetic disorders, such as thalassaemia, may be explained by the relative rarity of these conditions individually, the (already strained) resources of such groups and also by the somewhat ambiguous character of carrier status. Carrier status has been described by Timmermans and Buchbinder (2010) as analogous to a liminal state, a halfway house between health and disease, the significance of which can be difficult to interpret. Carrier couples may struggle to reconcile this ambiguity in their identities which, in turn, may render them alienated from such groups, the ethos of which may also clash with their own reactions to their genetic status.

## 12 RESEARCH RECOMMENDATIONS

Further research is indicated to explore understandings of, and reactions to, genetic risk among the general population, particularly in relation to different types of genetic screening programme (pre‐conceptual, prenatal, newborn) and for contrasting conditions. Anticipating (and responding appropriately to) the information and support needs of the screened population has been described as paramount to the successful implementation of screening programmes. This is especially the case, as has been demonstrated by this study and others (e.g., Evers‐Kiebooms, Denayer, & Berghe, 1990; Hershberger et al., 2012), where information needs fluctuate across the couple's decision‐making trajectory and use of genetic technologies is inconsistent across pregnancies (e.g., Jahida). Unlike screening for non‐heritable disorders like Down's Syndrome where a positive result is typically a “one off” event (and therefore contact with the support group may be fleeting), genetic risk for conditions like thalassaemia recurs (assuming the same reproductive partner for each pregnancy), suggesting a need for ongoing forms of information and support that are tailored to its unique, and expanding, challenges.

Additional research could also explore the format and content of educational resources around genetic disease that people undergoing genetic carrier screening find most useful at different time points in their reproductive journey. Indeed, at a time when panel screens for a range of genetic conditions are emerging on the horizon of standard NHS antenatal care (Nuffield Council on Bioethics, 2017p. 84), a greater understanding of the way such information is processed and formulated into conceptualisations of different genetic disorders, as well as responses to genetic risk, is now of paramount importance.

## 13 STUDY LIMITATIONS

Recruiting participants through the UKTS may have introduced bias to the sample as its members have typically received a diagnosis of thalassaemia in the family. Indeed, all four participants who discovered their thalassaemia carrier status through prenatal screening went on to have affected children. The perspectives of those who received a negative prenatal test result, or who terminated their pregnancy following a positive result, therefore, are missing. Although such individuals may be recruited through antenatal clinics, there are ethical concerns with undertaking interviews in the (typically highly strained and short) time between receiving a positive screening result and undergoing a prenatal diagnosis/selective pregnancy termination. However, as the focus of this analysis was not on the *outcomes *of the reproductive views and decisions per se, but rather on the *processes *and *resources* with which participants arrived at them, it was felt that the exclusion of these participants did not detract significantly from the utility of its findings.

## INFORMED CONSENT

All procedures followed were in accordance with the ethical standards of the responsible committee on human experimentation (institutional and national) and with the Helsinki Declaration of 1975, as revised in 2000 (5). Informed consent was obtained from all patients for being included in the study.

## AUTHORSHIP CONTRIBUTIONS

Dr Felicity Boardman designed the study, was responsible for the study set up and oversight of the data collection and led on both the data analysis, table creation and paper writing. She also provided final approval for manuscript submission. Dr Rachel Hale conducted all of the interviews, contributed to the interpretation and analysis of the data, and provided critical input to the writing of the paper. She also provided approval for manuscript submission.

## CONFLICTS OF INTEREST

Dr Felicity Boardman and Dr Rachel Hale have no conflicts of interest to declare.

## References

[jgc41042-bib-0001] Ahmed, S. , Bryant, L. , & Hewison, J. (2007). Balance is in the eye of the beholder: Providing information to support informed choices in antenatal screening via Antenatal Screening Web Resource. Health Expectations, 10(4), 309–320. 10.1111/j.1369-7625.2007.00455.x 17986068 PMC5060415

[jgc41042-bib-0002] Anido, A. , Carlson, L. M. , Taft, L. , & Sherman, S. L. (2005). Women’s attitudes toward testing for fragile X carrier status: A qualitative analysis. Journal of Genetic Counseling, 14(4), 295–306. 10.1007/s10897-005-1159-6 16047092

[jgc41042-bib-0003] Archibald, A. , Jaques, A. M. , Wake, S. , Collins, V. R. , Cohen, J. , & Metcalfe, S. A. (2009). ‘It’s something I need to consider’: Decisions about carrier screening for fragile X syndrome in a population of non‐pregnant women. American Journal of Medical Genetics, Part A, 149A, 2731–2738. 10.1002/ajmg.a.33122 19938084

[jgc41042-bib-0004] Archibald, A. , Smith, M. J. , Burgess, T. , Scarff, K. L. , Elliott, J. , Hunt, C. E. , … Amor, D. J. (2018). Reproductive genetic carrier screening for cystic fibrosis, fragile X syndrome, and spinal muscular atrophy in Australia: Outcomes of 12,000 tests. Genetics in Medicine, 20(5), 513–523. 29261177 10.1038/gim.2017.134

[jgc41042-bib-0006] Baillergeau, E. , & Duyvendak, J. W. (2016). Experiential knowledge as a resource for coping with uncertainty: Evidence and examples from the Netherlands. Health, Risk & Society, 18(7–8), 407–426. 10.1080/13698575.2016.1269878

[jgc41042-bib-0008] Barter, B. , Hastings, R. , Williams, R. , & Huws, J. (2016). Perceptions and discourses relating to genetic testing: Interviews with people with Down’s syndrome. Journal of Applied Research in Intellectual Disabilities, 1–12. 10.1111/jar.12256 27168113

[jgc41042-bib-0009] Beard, C. , Amor, D. J. , Di Pietro, L. , & Archibald, A. (2016). ‘I’m healthy, it’s not going to be me’: Exploring experiences of carriers identified through a population reproductive genetic carrier screening panel in Australia. American Journal of Medical Genetics. 10.1002/ajmg.a.37697 27150953

[jgc41042-bib-0010] Boardman, F. (2011). Negotiating discourses of maternal responsibility, disability and reprogenetics In Lewiecki‐Wilson C., & Cellio J. (Eds.), Disability and mothering: Liminal spaces of embodied knowledge (pp. 34-49). New York, NY: Syracuse University Press.

[jgc41042-bib-0011] Boardman, F. (2014). Knowledge is power? The role of experiential knowledge in genetically ‘risky’ reproductive decisions. Sociology of Health & Illness, 36(1), 137–150. 10.1111/1467-9566.12048 24111508

[jgc41042-bib-0012] Boardman, F. (2017). Experience as knowledge: Disability, distillation and (reprogenetic) decision‐making. Social Science & Medicine, 191, 186–193. 10.1016/j.socscimed.2017.09.013 28926777 PMC7610975

[jgc41042-bib-0013] Boardman, F. , Young, P. , & Griffiths, F. (2017). Impairment experiences, identity and attitudes towards genetic screening: The views of people with spinal muscular atrophy. Journal of Genetic Counseling, 10.1007/s10897-017-0122-7 PMC5794814 28664217

[jgc41042-bib-0014] Boardman, F. , Young, P. , Warren, O. , & Griffiths, F. (2017). The role of experiential knowledge within attitudes towards genetic carrier screening: A comparison of people with and without experience of Spinal Muscular Atrophy. Health Expectations, 00, 1–11. 10.1111/hex.12602 PMC5750730 28703871

[jgc41042-bib-0015] Braido, F. , Baiardini, I. , Sumberesi, M. , Canonica, G. W. , Blasi, F. , & Castellani, C. (2015). Public awareness on cystic fibrosis: Results from a national pragmatic survey. European Respiratory Journal, 46, 246–267. 10.1183/09031936.00221014 25882806

[jgc41042-bib-0016] Chen, E. , & Schiffman, J. (2000). Attitudes toward genetic counseling and prenatal diagnosis among a group of individuals with disabilities. Journal of Genetic Counseling, 9(2), 137–152. 12530456 10.1023/a:1009412025722

[jgc41042-bib-0017] Cousens, N. E. , Gaff, C. L. , Metcalfe, S. , & Delatycki, M. B. (2010). Carrier screening for beta‐thalassaemia: A review of international practice. European Journal of Human Genetics, 18, 1077–1083. 10.1038/ejhg.2010.90 20571509 PMC2987452

[jgc41042-bib-0018] Davies, S. (2016). Annual report of the Chief Medical Officer 2016: Generation genome. Retrieved from https://www.gov.uk/government/publications/chief-medical-officer-annual-report-2016-generation-genomeaccessed09/11/17

[jgc41042-bib-0019] De Wolfe, P. (2002). Private tragedy in social context? Reflections on disability, illness and suffering. Disability & Society, 17(3), 255–267. 10.1080/09687590220139847

[jgc41042-bib-0020] Denier, Y. (2014). From brute luck to option luck? On genetics, justice and moral responsibility in reproduction In Beauchamp T., LeRoy W., Kahn J., & Mastroianni A. (Eds.), Contemporary issues in bioethics. New York, NY: Wadsworth. 10.1093/jmp/jhq007 20181644

[jgc41042-bib-0021] Downs Syndrome Association (2017). About Down’s syndrome: Prenatal screening FAQs. Retrieved from https://www.downs-syndrome.org.uk/about/pre-natal-faqs/accessed30/11/17

[jgc41042-bib-0022] Etchegary, H. , Potter, B. , Howley, H. , Cappelli, M. , Coyle, D. , Graham, I. , Walker, M. , et al. (2008). The influence of experiential knowledge on prenatal screening and testing decisions. Genetic Testing, 12(1), 115–124. 10.1089/gte.2007.0057 18307384

[jgc41042-bib-0023] Evers‐Kiebooms, G. , Denayer, L. , & Van den Berghe, H. (1990). A child with cystic fibrosis: II. Subsequent family planning decisions, reproduction and use of prenatal diagnosis. Clinical Genetics, 37(3), 207–215. 2323090 10.1111/j.1399-0004.1990.tb03504.x

[jgc41042-bib-0024] Fanos, J. , Spangner, K. , & Musci, T. (2006). Attitudes towards prenatal screening and testing for fragile X. Genetics in Medicine, 8, 129–133. 10.1097/01.gim.0000200158.66554.7f 16481897

[jgc41042-bib-0025] Gibbs, G. (2007). Analyzing qualitative data. London, UK: Sage Publications Ltd.

[jgc41042-bib-0026] Goffman, E. (1963). Stigma: Notes on the management of a spoiled identity. London, UK: Penguin.

[jgc41042-bib-0028] Gregg, A. , Van der Veyver, I. B. , Gross, S. , Madankumar, R. , Rink, B. D. , & Norton, M. E. (2014). Noninvasive prenatal screening by next‐generation sequencing. Annual Review of Genomics and Human Genetics, 15, 327–347. 10.1146/annurev-genom-090413-025341 24849140

[jgc41042-bib-0029] Hasegawa, L. E. , Fergus, K. A. , Ojeda, N. , & Au, S. M. (2011). Parental attitudes towards ethical and social issues surrounding the expansion of newborn screening using new technologies. Public Health Genomics, 14(4–5), 298–306. 20689248 10.1159/000314644 PMC3214890

[jgc41042-bib-0030] Henneman, L. , Borry, P. , Chokoshvili, D. , Cornel, M. C. , van EI , C. G. , … A. , ESHG . (2016). Responsible implementation of expanded carrier screening. Summary and recommendations of the European Society of Human Genetics. European Journal Human Genetics, 24(6), e1–e12. 10.1038/ejhg.2015.271 PMC4867464 26980105

[jgc41042-bib-0031] Hershberger, P. E. , Gallo, A. M. , Kavanaugh, K. , Olshansky, E. , Schwartz, A. , & Tur‐Kaspa, I. (2012). The decision‐making process of genetically at‐risk couples considering preimplantation genetic diagnosis: Initial findings from a grounded theory study. Social Science and Medicine, 74(10), 1536–1543. 10.1016/j.socscimed.2012.02.003 22445765 PMC3328546

[jgc41042-bib-0032] Hickman, M. , Modell, B. , Greengross, P. , Chapman, C. , Layton, M. , Falconer, S. , & Davies, S. C. (1999). Mapping the prevalence of sickle cell and beta thalassaemia in England: Estimating and validating ethnic‐specific rates. British Journal of Haematology, 104(4), 860–867. 10.1046/j.1365-2141.1999.01275.x 10192451

[jgc41042-bib-0033] Human Genetics Commission (2011). Increasing options, informing choice: A report on preconception genetic testing and screening. Retrieved from https://f.hypotheses.org/wp-content/blogs.dir/257/files/2011/04/2011.HGC_.-Increasing-options-informing-choice-final2.pdfaccessed28/11/17

[jgc41042-bib-0034] Ioannou, L. , Delatycki, M. B. , Massie, J. , Hodgson, J. , & Lewis, S. (2015). ‘Suddenly having two positive people who are carriers is a whole new thing’ – Experiences of couples both identified as carriers of cystic fibrosis through a population‐based carrier screening program in Australia. Journal of Genetic Counseling, 24(6), 987–1000. 10.1007/s10897-015-9833-9 25925605

[jgc41042-bib-0035] Ioannou, L. , Massie, J. , Lewis, S. , McClaren, B. , Collins, V. , & Delatycki, M. B. (2014). ‘No thanks’ – Reasons why pregnant women declined an offer of cystic fibrosis carrier screening. Journal of Community Genetics, 5, 109–117. 10.1007/s12687-013-0151-3 23715719 PMC3955462

[jgc41042-bib-0036] Janssens, S. , Chokoshvilli, D. , Binst, C. , Mahieu, I. , Henneman, L. , De Paepe, A. , & Borry, P. (2016). Attitudes of cystic fibrosis patients and parents toward carrier screening and related reproductive issues. European Journal of Human Genetics, 24, 506–512. 10.1038/ejhg.2015.160 26220700 PMC4929879

[jgc41042-bib-0038] Katz Rothman, B. (1986). The tentative pregnancy: Prenatal diagnosis and the future of motherhood. New York, NY: Viking.

[jgc41042-bib-0039] Kay, E. , & Kingston, H. (2002). Feelings associated with being a carrier and characteristics of reproductive decision making in women known to be carriers of X linked conditions. Journal of Health Psychology, 7(2), 169–181. 10.1177/1359105302007002456 22114236

[jgc41042-bib-0040] Kelly, S. (2009). Choosing not to choose: Reproductive responses of parents of children with genetic conditions or impairments. Sociology of Health & Illness, 31(1), 81–97. 10.1111/j.1467-9566.2008.01110.x 19144088

[jgc41042-bib-0041] Kenen, R. (1994). The Human Genome Project: Creator of the potentially sick, potentially vulnerable and potentially stigmatised? In Robinson L. (Eds.), Life and death under high technology medicine. Manchester, UK: Manchester University Press.

[jgc41042-bib-0042] Lippman, A. (1999). Embodied knowledge and making sense of prenatal diagnosis. Journal of Genetic Counseling, 8(5), 255–274. 10.1023/A:1022901131305 26142373

[jgc41042-bib-0043] Locock, L. , & Kai, J. (2008). Parents’ experiences of universal screening for haemoglobin disorders: Implications for practice in a new genetics era. British Journal of General Practice, 58, 161–168. 10.3399/bjgp08X277276 PMC2249791 18318970

[jgc41042-bib-0045] Markens, S. , Browner, C. , & Preloran, M. H. (2010). Interrogating the dynamics between power, knowledge and pregnant bodies in amniocentesis decision‐making. Sociology of Health and Illness, 32(1), 37–56. 10.1111/j.1467-9566.2009.01197.x 19891618

[jgc41042-bib-0046] McClaren, B. , Delatycki, M. B. , Collins, V. , Metcalfe, S. A. , & Aitken, M. (2008). ‘It is not in my world’: An exploration of attitudes and influences associated with cystic fibrosis carrier screening. European Journal of Human Genetics, 16(4), 435–444. 10.1038/sj.ejhg.5201965 18059419

[jgc41042-bib-0047] Middleton, A. , Hewison, J. , & Mueller, R. (1998). Attitudes of deaf adults towards genetic testing for hereditary deafness. American Journal of Human Genetics, 63(4), 1175–1180. 10.1086/302060 9758618 PMC1377492

[jgc41042-bib-0048] Middleton, A. , Morley, K. , Bragin, E. , Firth, H. V. , Hurles, M. E. , Wright, C. F. , & Parker, M. (2016). Attitudes of nearly 7000 health professionals, genomic researchers and publics toward the return of incidental results from sequencing research. European Journal Human Genetics, 24, 21–29. 10.1038/ejhg.2015.58 PMC4795240 25920556

[jgc41042-bib-0051] Moultrie, R. R. , Kish‐Doto, J. , Peay, H. , & Lewis, M. A. (2016). A review on spinal muscular atrophy: Awareness, knowledge and attitudes. Journal of Genetic Counseling, 25(5), 892–900. 10.1007/s10897-016-9955-8 27084745

[jgc41042-bib-0052] Nazareth, S. B. , Lazarin, G. A. , & Goldberg, J. D. (2015). Changing trends in carrier screening for genetic disease in the United States. Prenatal Diagnosis, 35, 931–935. 10.1002/pd.4647 26138560 PMC4758394

[jgc41042-bib-0053] Nuffield Council on Bioethics . (2017). Non‐invasive prenatal testing: Ethical issues. Retrieved from http://nuffieldbioethics.org/project/non-invasive-prenatal-testingaccessed30/11/17

[jgc41042-bib-0054] Plantinga, M. , Erwin, B. , Abbott, K. M. , Sinke, R. J. , Lucassen, A. M. , Schuurmans, J. , … van Langen, I. M. (2016). Population‐based preconception carrier screening: How potential users from the general population view a test for 50 serious diseases. European Journal Human Genetics, 1–7. 10.1038/ejhg.2016.43 27165008 PMC5027688

[jgc41042-bib-0055] Prior, T. , Snyder, P. , Rink, B. D. , Pearl, D. K. , Pyatt, R. E. , Mihal, D. C. , … Garner, S. (2010). Newborn and carrier screening for spinal muscular atrophy. American Journal of Medical Genetics, Part A, 152A, 1608–1616. 10.1097/OGX.0b013e318202208f 20578137

[jgc41042-bib-0057] Raspberry, K. , & Skinner, D. (2010). Enacting genetic responsibility: Experiences of mothers who carry the fragile X gene. Sociology of Health and Illness, 33(3), 420–433. 10.1111/j.1467-9566.2010.01289.x 21054442 PMC3057279

[jgc41042-bib-0058] Reed, K. (2011). He’s the dad isn’t he? Gender, race and the politics of prenatal screening. Ethnicity & Health, 16(4–5), 327–341. 10.1080/13557858.2010.531196 21797721

[jgc41042-bib-0059] Roadhouse, C. , Shuman, C. , Anstey, K. , Sappleton, K. , Chitayat, D. , & Ignagni, F. (2018). Disability experiences and perspectives regarding reproductive decisions, parenting and the utility of genetic services: A qualitative study. Journal of Genetic Counseling, 27, 1360–1373. 10.1007/s10897-018-0265-1 29909595

[jgc41042-bib-0061] Rothwell, E. , Anderson, R. A. , Swoboda, K. , Stark, L. , & Botkin, J. R. (2013). Public attitudes regarding a pilot study of newborn screening for spinal muscular atrophy. American Journal of Medical Genetics, Part A, 161(4), 679–686. 10.1002/ajmg.a.35756 PMC3606635 23443997

[jgc41042-bib-0062] Roy, T. , & Chatterjee, S. C. (2007). The experiences of adolescents with thalassaemia in West Bengal. India, Qualitative Health Research, 17(1), 85–93. 17170246 10.1177/1049732306296400

[jgc41042-bib-0063] Shakespeare, T. (2006). Disability rights and wrongs. London, UK: Sage.

[jgc41042-bib-0064] Shaw, A. , & Hurst, J. A. (2006). What is this genetics anyway? Understandings of genetics, illness causality and inheritance among British Pakistani users of genetics services. Journal of Genetic Counseling, 17, 373–383. 10.1007/s10897-008-9156-1 18607703

[jgc41042-bib-0067] Tarini, B. A. , & Goldenberg, A. J. (2012). Ethical issues with newborn screening in the genomics era. Annual Review of Genomics, 13, 381–393. 10.1146/annurev-genom-090711-163741 PMC3625041 22559326

[jgc41042-bib-0068] Telfer, P. (2009). Update on survival in thalassaemia major. Haemoglobin, 33(sup1), S76–S80. 10.3109/03630260903347336 20001636

[jgc41042-bib-0069] Timmermans, S. , & Buchbinder, M. (2010). Patients in waiting: Living between sickness and health in the genomics era. Journal of Health and Social Behaviour, 51(4), 408–423. 10.1177/0022146510386794 21131618

[jgc41042-bib-0070] Tsianakas, V. , Atkin, K. , Calnan, M. , Dormandy, E. , & Marteau, T. (2011). Offering antenatal sickle cell and thalassaemia screening to pregnant women in primary care: A quality study of women’s experiences and expectations of participation. Health Expectations, 15, 115–125. 10.1111/j.1369-7625.2011.00669.x 21366810 PMC5060612

[jgc41042-bib-0071] Van der Wal, J. T. , Martin, L. , Manniën, J. , Verhoeven, P. , Hutton, E. K. , & Reinders, H. S. (2015). A qualitative study of how Muslim women of Moroccan descent approach to antenatal screening. Midwifery, 31, e43–e49. 10.1016/j.midw.2014.12.007 25592301

[jgc41042-bib-0072] Watson, E. K. , Williamson, R. , & Chapple, J. (1991). Attitudes to carrier screening for cystic fibrosis: A survey of health care professionals, relatives of sufferers and other members of the public. British Journal of General Practice, 41, 237–240. PMC1371586 1931202

[jgc41042-bib-0073] Wright, K. , Bryant, L. , Morley, S. , Hewison, J. , Duff, A. , & Peckham, D. (2015). Presenting life with cystic fibrosis: A Q‐methodological approach to developing balanced, experience‐based prenatal screening information. Health Expectations, 18(5), 1349–1432. 10.1111/hex.12113 23910894 PMC5060888

[jgc41042-bib-0074] Ziebland, S. , & Herxheimer, A. (2008). How patients’ experiences contribute to decision‐making: Illustrations from DIPex (personal experiences of health and illness). Journal of Nursing Management, 16, 433–439. 10.1111/j.1365-2834.2008.00863.x 18405260

